# Hyaluronate-Functionalized Graphene for Label-Free Electrochemical Cytosensing

**DOI:** 10.3390/mi9120669

**Published:** 2018-12-18

**Authors:** Aihua Jing, Chunxin Zhang, Gaofeng Liang, Wenpo Feng, Zhengshan Tian, Chenhuan Jing

**Affiliations:** 1 School of Medical Technology and Engineering, Henan University of Science and Technology, Luoyang 471023, China; zhchunx1@haust.edu.cn (C.Z.); fwp238@haust.edu.cn (W.F.); 2 Medical College, Henan University of Science and Technology, Luoyang 471023, China; 3 School of Chemistry and Chemical Engineering, Pingdingshan University, Pingdingshan 467000, China; 180318220790@stu.haust.edu.cn; 4 Pingdingshan No. 1 Middle School, Pingdingshan 467000, China; 16jingchenh@haust.edu.cn

**Keywords:** biocompatible interface, graphene oxide, colorectal cancer cells HCT-116, electrochemical impedance spectroscopy

## Abstract

Electrochemical sensors for early tumor cell detection are currently an important area of research, as this special region directly improves the efficiency of cancer treatment. Functional graphene is a promising alternative for selective recognition and capture of target cancer cells. In our work, an effective cytosensor of hyaluronate-functionalized graphene (HG) was prepared through chemical reduction of graphene oxide. The as-prepared HG nanostructures were characterized with Fourier transform infrared spectroscopy and transmission electron microscopy coupled with cyclic voltammograms and electrochemical impedance spectroscopy, respectively. The self-assembly of HG with ethylene diamine, followed by sodium hyaluronate, enabled the fabrication of a label-free electrochemical impedance spectroscopy cytosensor with high stability and biocompatibility. Finally, the proposed cytosensor exhibited satisfying electrochemical behavior and cell-capture capacity for human colorectal cancer cells HCT-116, and also displayed a wide linear range, from 5.0 × 10^2^ cells∙mL^−1^ to 5.0 × 10^6^ cells∙mL^−1^, and a low detection limit of 100 cells∙mL^−1^ (S/N = 3) for quantification. This work paves the way for graphene applications in electrochemical cytosensing and other bioassays.

## 1. Introduction

Studying intact living cells efficiently using electrochemical sensing systems has fundamental significance and practical importance in biotechnology, biological device design, drug discovery, molecular medicine, and disease diagnosis. Various electrochemical sensors have been devoted to the design and fabrication of specific nanostructured biointerfaces with high conductivity and good cell adhesion for the detection of cell viability [1]. The current electrical nanostructured biointerfaces built of cytosensors have attracted considerable attention in measuring the expression of glycans on single cells [2] and immobilizing antibodies for rapid diagnosis [3], as it can clearly separate the surface bindings, easily quantify signals, and obtain a high sensitivity. Wightman et al. [4] stimulated cells by carbon fiber electrodes to release the neurotransmitter catecholamine, which was detected by converting the chemical signal to the output of the electrical signal. Giaever group [5] designed a nanoscale gold electrode biosensor based on cell adhesion growth characteristics. When the cell was attached to the electrode, the change of the interface impedance was detected along the cell changes. Zhu groups [6] immobilized carboxymethyl chitosan-functionalized graphene for a label-free electrochemical impedance spectroscopy cytosensor and detected HL-60 cells by an electrochemical technique. Damiati [7] presented an efficient acoustic and hybrid three-dimensional (3D)-printed electrochemical biosensor for the detection of HepG2 cells. Various rapid and label-free biomarker detection techniques, including electronic [8,9], optical [10,11,12,13], and mechanical [14,15,16,17], have been developed to detect cancer specific protein biomarkers, as well as to measure the specific gravity of food samples in real-time [18]. Bioelectronic noses are skilled at detecting odorant molecules and compounds at low detection limits, with high selectivity and sensitivity [19,20,21], and will be attractive in the fields of cancer detection [22,23]. Other non-invasive techniques, such as proton-transfer-reaction mass spectrometry [24] and selected ion flow tube mass spectrometry [25], have been developed based on the analysis of human volatilome for medical diagnostics and for monitoring of the patients’ health [26,27]. Consequently, there is still an increasing desire to develop new novel cytosensors for detection of cells with dimensional compatibility, high sensitivity, simplicity, selectivity, and low cost. 

Graphene has attracted considerable interest for its remarkable structures, electronic properties, and tunable surface functionalities [18]. The biofunctionalized nanocomposites of graphene have been applied in the fabrication of biosensors, such as DNA [28] and microRNA detection [29], small molecules [30,31,32], antigen detection [33], and living cell studies [29,34]. A table showing the advantages and disadvantages of graphene and functionalized graphene is shown in Table S1. However, the surface of graphene oxide is not suitable for constructing interfaces with large human cells for the incompatible dimensions. Hyaluronic acid (HA) widely exists in extracellular matrix and is a naturally occurring mucopolysaccharide that has been extensively designed in biomedical systems for its biocompatibility, biodegradability, and non-immunogenicity [35]. Meanwhile, studies have shown the surfaces of some tumor cells are rich in hyaluronic acid receptor protein CD44 [36]. Thus, the obtained hyaluronate-functionalized graphene (HG) composite can achieve detection of differential tumor cells by hyaluronic acid receptors on the surface, as desirable for biomedical applications.

In this paper, a composite of HG was prepared and displayed suitability for assembling on the surface of a glass carbon electrode (GCE) to a cytosensor. Accordingly, HA can express target hyaluronic receptors of high affinity, specifically, while some free carboxylic groups on the HA molecular chains cross-linked with other residual groups can act as linkers between the graphene sheets and cells. In addition, we have chosen the colorectal cancer cell HCT-116 to be detected by the as-prepared cytosensors.

The as-prepared cytosensor showed better stability and sensitivity than that of non-HG sensor, thus offering the potential of graphene for the construction of cytosensing and other impedance cell device.

## 2. Materials and Methods

### 2.1. Materials and Apparatus

Graphene oxide was prepared in our lab and ultrasonicated to 1.0 mg∙mL^−1^. Sodium hyaluronate was obtained from Sangon biotech (Shanghai, China). N-hydroxysuccinimide (NHS) and 1-(3-dimethylaminopropyl)-3-ethylcarbodiimide hydrochloride (EDC) were obtained from Tianjin BASF chemical company (Tianjin, China). All other reagents were of analytical grade and used as received without further purification, unless otherwise specified. Phosphate-buffer solutions (PBS) was prepared by mixing solutions of NaH_2_PO_4_ and Na_2_HPO_4_ and then adjusted with 0.1 mol∙L^−1^ NaOH or H_3_PO_4_. All solutions were prepared with Milli-Q water. 

Fourier transform infrared (FTIR) spectra were taken at room temperature from 400 cm^−1^ to 4000 cm^−1^ using a Nicolet IS10 FTIR (Thermo Fisher Company, Waltham, MA, USA). The morphologies were characterized by a transmission electron microscope (TEM, JEOL JTM-2100, Tokyo, Japan). Microscopy images were obtained with Nikon Ti-S inverted microscopy (Nikon Corporation, Tokyo, Japan).

### 2.2. Synthesis of Hyaluronate-Functionalized Graphene (HG)

Firstly, EDC was added to the buffer solution of 2-(N-morpholine)ethenylsulfonic acid (MES buffer solution, pH = 6.0) [36] and stirred for 30 min at room temperature before 5.0 mg NHS was added and stirred for 2 h. The product was moved to 100 mL volumetric flask to avoid light preservation, and activated solution of carboxyl group was received. One hundred milliliters of hyaluronic acid aqueous solution (1.0 mg∙mL^−1^) was poured into 500 mL bottle at room temperature. Under magnetic stirring condition, nitrogen was added to the solution for 30 min to remove oxygen in the reaction system. One hundred milliliters of amino group functionalized graphene oxide (NH_2_/GO) made from GO powder (1.0 mg∙mL^−1^), as reported in Reference [37], and the above carboxyl group activation solution were slowly added into the above solution and stirred at 30 °C for 4 h. the entire process was conducted under continuous flow of nitrogen. The mixture was then treated in excess acetone and dialyzed by distilled water for 2 days using a dialysis membrane (MWCO 25 kDa, Shanghai, China) to remove unreacted reagents. Thereafter, HG was obtained [6]. 

### 2.3. Electrode Fabrication

**Figure 1:**
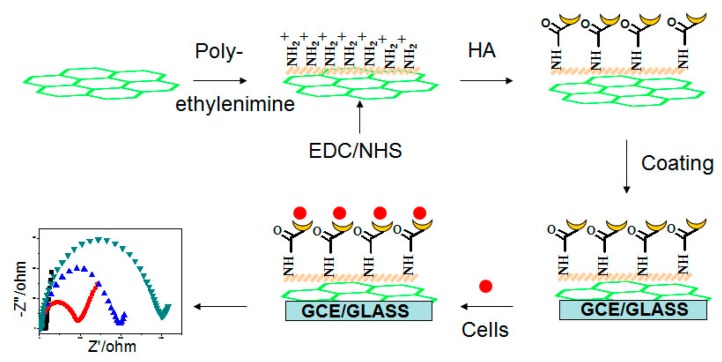
Procedure for preparing electrochemical cytosensor. EDC–1-(3-dimethylaminopropyl)-3-ethylcarbodiimide hydrochloride, NHS–N-hydroxysuccinimide, GCE– glass carbon electrode, HA–Sodium hyaluronate.

The GCE was mirror-polished with 0.3 μm and 0.05 μm alumina slurries (Beuhler), followed by sonication in acetone, ethanol, and pure water. The electrode was dried at room temperature before 10 μL of mixture of HG solution and 1% Nafion (volume ratio 20:1) was dropped on the pretreated GCE to fabricate HG/GCE. It was rinsed 3 times in PBS solution (pH = 7.4) and placed in a clean drying box. The fabrication steps are shown in Figure 1.

### 2.4. Cell Culture and Maintenance

Human colorectal cancer cells HCT116 and NIH/3T3, obtained from the American Type Culture Collection, were maintained in MacCoy’s 5A medium (Invitrogen, Carlsbad, CA USA) supplemented with 10% fetal bovine serum (FBS) (GIBCOBRL Laboratories, NY, USA), 2.0 × 10^−3^ mol∙L^−1^ L-glutamic acid, and 1% penicillin-streptomycin solution (Sigma Chemical Co., St. Louis, MO, USA) in a humidified incubator at 37 °C in an atmosphere of 5% CO_2_ (Thermo Scientific; Waltham, MA, USA). The medium was changed every 3 days, and cells were pass aged using Trypsin/EDTA.

### 2.5. Cell Immobilization

Cells were separated from medium by centrifugation at 900 rpm for 3 min at the growth retardation stage (3 days), and washed twice with sterile PBS (pH = 7.4). The sediments were resuspended in PBS to obtain cell suspension with a final concentration of 5.0 × 10^5^ cells∙mL^−1^, determined by a Hemocytometer. Two microliters of cell suspension were dropped on the HG/GCE and incubated at 37 °C for 2 h for successful immobilization on the electrodes.

### 2.6. Electrochemical Measurements

All electrochemical measurements were performed on a CHI660E electrochemical workstation (Chenhua, Shanghai, China), with a conventional three-electrode system comprised of a platinum wire auxiliary, a saturated calomel reference, and a modified HG/GCE. Solutions were degassed with nitrogen to remove O_2_. Electrochemical impedance spectroscopy (EIS) experiments were performed in a 2.0 × 10^−3^ mol∙L^−1^ K_3_Fe(CN)_6_/K_4_Fe(CN)_6_ (1:1) mixture with 0.1 mol∙L^−1^ KCl as supporting electrolyte, with an alternating current voltage of 5.0 mV and the frequency range of 0.01–100 kHz.

## 3. Results and Discussion

### 3.1. Fabrication of the Cytosensor

**Figure 2:**
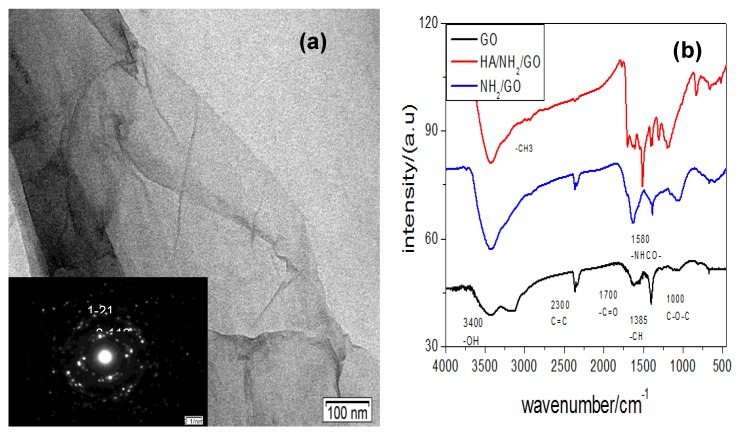
(**a**) TEM diagram of hyaluronate-functionalized graphene (HG), and (**b**) FTIR spectra of graphene oxide (GO), NH_2_/GO, and HG.

HG showed a good dispensability in water due to hydrophilicity of hyaluronic acid modification on its surface. Figure 2a shows morphology of the as-prepared HG. From it we found film-like shapes with little rippled wrinkles. Its inset shows corresponding selected area electron diffraction (SAED) pattern. The well-defined diffraction spots and rings in the pattern were confirmed by the typical hexagonal lattice of crystalline carbon in HG. 

We then designed a cytosensing platform of HG interface for the immobilization and sensitive electrochemical detection of cancer cells. In the fabrication of electrochemical sensors, ethylene diamines with negative charge acted as the binding linker between HA and GO through formation of amide bond. The GO was fabricated with HA and monitored by FTIR spectra, as shown in Figure 2b. The GO curve displayed a characteristic intense peak at 1730 cm^−1^ (the C=O vibration), a broad peak centered at 3350 cm^−1^ (the O-H vibration) [38], a peak at 1220 cm^−1^ (the C-OH stretching), and a C-O stretching peak at 1050 cm^−1^ [38]. After ethylene diamine was grafted onto the GO, a characteristic peak at 1575 cm^−1^ (N-H bending mode) appeared in curve NH_2_–GO [39], confirming that NH_2_ had been grafted onto the surface of GO. The conjugation of HA and NH_2_/GO was confirmed through amidation from the COOH groups in the HA and NH_2_ groups in HG, as shown in curve HA–GO. A new peak at 1660 cm^−1^ was observed and attributed to the presence of CO–NH groups in the HG composite [40]. This further demonstrated that the electrostatic adsorption of HA onto NH_2_/GO was substantial. 

### 3.2. HG Film for Adhesion of HCT-116 Cells

**Figure 3:**
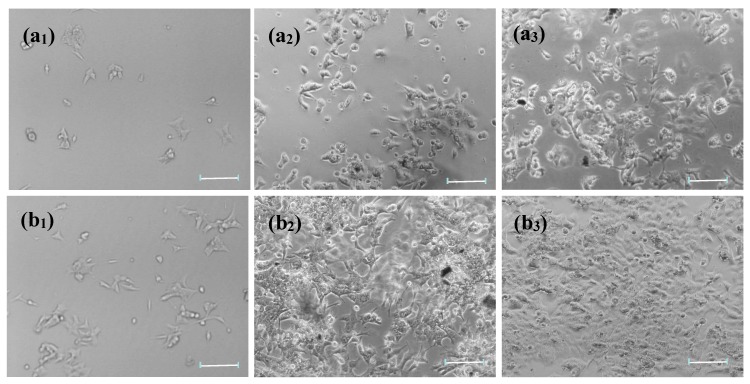
Microscopy images of HCT-116 cells proliferated on (**a**) bare glass and (**b**) HG/glass for (**a_1_**, **b_1_**) 12 h, (**a_2_**,**b_2_**) 24 h, and (**a_3_**,**b_3_**) 48 h respectively. Scale bar = 100 μm.

Figure 3 shows microscopic images of HCT-116 cells proliferated on bare glass and HG/glass for 12h, 24 h, and 48 h. The photos of HG/glass displayed a highly immobilized density of HCT-116 cells compared to that of bare glass. These results indicated that the immobilization capacity for cells in the biocompatible interface of assembled HG was highly improved. Moreover, the density of HCT-116 cells immobilized on the HG film increased noticeably from 12 h (b_1_), to 24 h (b_2_), and to 48 h (b_3_), in which HCT-116 cells were spread to irregular shapes cover all surface of HG film. The cells were alive, as evidenced by the morphology of the distinguishable filopodia, a good indicator of cell adhesion to material surfaces and cell viability [41]. The density of cells immobilized on the HG film increased with increased incubation time, suggesting that the HCT-116 cells were capable of not only adhering to HG film but also had a good viability on the film. This was due to HA receptors on HG cytosensors possibly recognizing the HCT-116 cells and maintaining the activity of cells. Moreover, the cell viability was detected by a scratch test (Figure S1) [42]. The HCT-116 cells had greater migration ability on the HG/Glass. From Figure S1a, in a scratch area on which no HCT-116 cell was observed in the center, the HCT-116 cells began to migrate toward the center of the scratch at 12 h. The HCT-116 cells migrated toward the center of the scratch more but did not fill the scratch by 24 h. Thus, the HG cytosensors did not show cytotoxicity and were suitable for preserving the activity of immobilized living cells with good biocompatibility and promoted cell adhesion and growth.

### 3.3. Electrochemical Characteristics of Cytosensor

**Figure 4:**
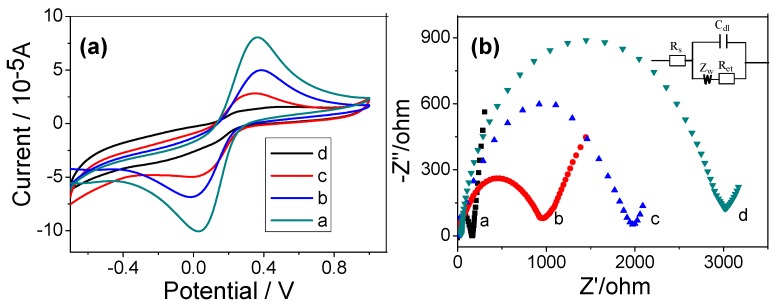
(**a**) Cyclic voltammograms and (**b**) Nyquist plots of impedance spectra at different electrodes in a solution of 1.0 × 10^−2^ mol∙L^−1^ phosphate-buffer solutions (PBS) (pH = 7.4) containing 2.0 × 10^−3^ mol∙L^−1^ K_3_Fe(CN)_6_-2.0 × 10^−3^ mol∙L^−1^ K_4_Fe(CN)_6_-0.1 mol∙L^−1^ KCl. Curve a, bare GCE; curve b, HG/ glass carbon electrode (GCE); curve c,d, HG/GCE after incubation with 5.0 × 10^2^ cells∙mL^−1^ and 5.0 × 10^3^ cells∙mL^−1^ for 40 min, respectively. Inset, displays the equivalent circuit applied in the work.

The cyclic voltammetric (CV) were carried out in a PBS (pH = 7.4) solution containing 0.1 mol∙L^−1^ KCl and 2.0 × 10^−3^ mol∙L^−1^ Fe(CN)_6_^3−^/Fe(CN)_6_^4−^ to investigate the properties of cytosensor. Figure 4a shows CVs of Fe(CN)_6_^3−^/Fe(CN)_6_^4−^ at the bare (curve a), HG (curve b), and cells/HG (curve c,d) modified GCE, respectively. The current of cathodic and anodic waves of HG modified GCE was lower than bare GCE, due to decelerated electron transfer [43,44]. After the HG modified electrode were incubated in HCT-116 cells (5.0 × 10^2^ cells∙mL^−1^ and 5.0 × 10^3^ cells∙mL^−1^) for 40 min, the peak currents for redox couple decreased successively because the cells obstructed the electron transfer kinetics of Fe(CN)_6_^3−^/Fe(CN)_6_^4−^.

The EIS measurement was more sensitive for monitoring changes in the surface features in the [Fe(CN)_6_]^3−^/[Fe(CN)_6_]^4−^probes system for the modified electrodes. The impedance spectra included two portions: One was a semicircle portion at higher frequencies that represented the electron transfer process, while the other was a linear portion at lower frequencies corresponding to the diffusion process. The semicircle diameter equals to the electron-transfer resistance (Ret) [45]. The charge transfer resistance change correlated with the impedance response of different assembled steps. Figure 4b shows the EIS of the electrode at different stages. For the bare GCE, the redox process of Fe(CN)_6_^3−^/Fe(CN)_6_^4−^ probe showed a very small semicircle at high frequencies and a Ret (the electron transfer impedance) value of 160 ± 5 Ω (curve a). After the HG was assembled on the electrode, the HG modified GCE showed a higher resistance (curve b), with the Ret value increasing to 913 ± 25 Ω, implying that the presence of HG lowers the conductivity due to numerous defects on the electrode. Subsequently, different concentrations of HCT-116 cells (5.0 × 10^2^ cells∙mL^−1^ and 5.0 × 10^3^ cells∙mL^−1^) were immobilized onto the electrode, and the Ret increased significantly to 1894 ± 50 Ω (curve c) and 2981 ± 95 Ω (curve d) as the concentration of the cells was increased, which was attributed to the fact that the poor conductivity of cells further hampered the redox probe of Fe(CN)_6_^3−^/Fe(CN)_6_^4−^ close to the surface of the electrode. The EIS results indicate that the increased impedence signal depended on the surface coverage of the cells which was directly proportional to the concentration of the cells used. Therefore, the HG modified electrode possibly can be used to detect target cells.

### 3.4. Sensitivity of the Cytosensor

The designed architecture of HG electrode provides a sensitive impedance cytosensor for adhesion and immobilization of HCT-116 cells. The selectivity of the cytosensor was evaluated. Two different cells were compared by monitoring the Ret value (Figure S2). When the HG/GCE was incubated with HCT-116 cells at concentration of 1.0 × 10^5^ cells∙mL^−1^, in the Ret value was observed to be much larger than when no cells were incubated. However, in the case of NIH/3T3 cells at the same concentration, a minor increase in Ret was observed, which was in accord with the microscopy results. A control test was also made with HG and NH_2_/GO modified GCE (Figure S3). The results showed that the Ret of HG/GCE was much higher than NH_2_/GO/GCE at the same HCT-116 cell concentrations. Therefore, it is reasonable that the cytosensor can detect HCT-116 cells.

**Figure 5:**
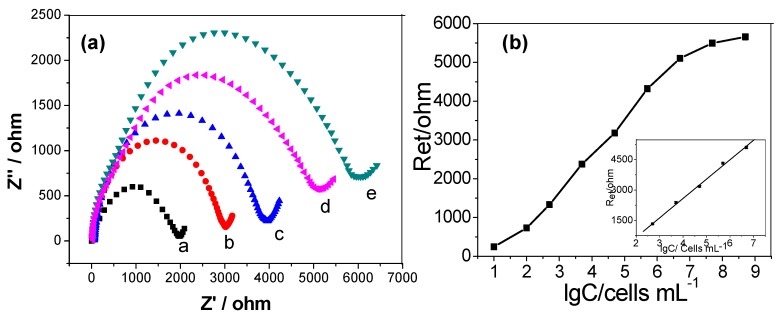
(**a**) Nyquist plots in 1.0 × 10^−2^ mol∙L^−1^ PBS (pH = 7.4) solution containing 0.1 mol∙L^−1^ KCl and 2.0 × 10^−3^ mol∙L^−1^ Fe(CN)_6_^3−^/Fe(CN)_6_^4−^ recorded on the Cells/HG/GCE with the cell concentrations of 5.0 × 10^2^ cells∙mL^−1^, 5.0 × 10^3^ cells∙mL^−1^, 5.0 × 10^4^ cells∙mL^−1^, 5.0 × 10^5^ cells∙mL^−1^ and 5.0 × 10^6^ cells∙mL^−1^. (**b**) Linear relationship between electron-transfer resistance and logarithm of HCT-116 cell concentrations. Inset is the calibration curve of the impedance sensor for determination of HCT-116 cells. Error bars represent standard deviation. Every point was an average value of three models of the cytosensors for independent measurements.

In order to evaluate the efficiency of the cytosensor, various concentrations of HCT-116 cells were modified on the HG/GCE. The obtained corresponding Nyquist plots of impedance spectra are shown in Figure 5a. From Figure 5a, it can be seen that with increasing concentration of HCT-116 cells, the diameter of the Nyquist circle clearly increased. This was due to insulation of cell membranes hindering the electron transfer. The inset shows the simulated Randle’s equivalent circuit. The modified equivalent circuit was in good agreement with the measurement system over the entire measurement frequency range. The changes of Ret value by modifications was much larger than those of other impedance elements and was a suitable probe for monitoring the changes of the assembly process. 

Figure 5b displays a linear relation between Ret responses and logarithmic values of cell concentrations in the range from 5.0 × 10^2^ cells∙mL^−1^ to 5.0 × 10^6^ cells∙mL^−1^. R_et_ = −1291.5 + 969.1lgC, with a correlation coefficient R of 0.993 (n = 5). The limit of detection calculated from the slope of the linear plot and the five times value of standard deviation was 100 cells∙mL^−1^ [46]. The proposed cytosensor exhibited a low detection limit for cancer cells, presumably because HA had great cell-capture ability towards HCT-116 cells, owing to the specific reaction between HA and CD44. Moreover, cytotoxicity was not detected by the HG/GCE cytosensor, thus resulting in the capture and detection of living cells. The impedance detection performances of the HCT-116 obtained at the HG/GCE were compared with other analogous modified electrodes or sensors previously reported in literature, as summarized in Table 1. It can be seen that the simplicity of our sensor was not affected the broad working linear range and low detection limit. 

### 3.5. Reproducibility and Stability of the Cytosensor

The reproducibility was estimated for five parallel determinations of 10^4^ cells∙mL^−1^ and 10^5^ cells∙mL^−1^ HCT-116 under the same experimental conditions. The relative standard deviation (RSD) was 6.78% and 7.21%, respectively, indicating acceptable precision and fabrication reproducibility. The EIS and CV response of the cytosensor had no obvious changes after a week in air at 4 °C. Moreover, the change in D_Ret_ for the same amount of HCT-116 cells (10^5^ cells∙mL^−1^) was less than 4.52%, indicating that the HG modified GCE possessed good stability and bioactivity. Therefore, the above results showed that the as-developed HG-based cytosensor could afford an applicable method for cancer cells detection and quantification with acceptable sensitivity, high stability, and good reproducibility.

## 4. Conclusions

In this work, an effective and reliable HG composite electrode with high dispersion and good biocompatibility was designed, which can be used to produce an electrochemical biosensor for label-free cancer cell detection. The composites showed high conductivity of graphene, while maintaining the good biocompatibility and adaptability of HA. The functional cytosensor uses HA receptor protein CD44 as a target to capture specific tumor cells (HCT-116) and has satisfactory performance of improved immobilization capacity for cells, as well as good biocompatibility for preserving the activity of immobilized living cells. The detection limit is 100 cells∙mL^−1^. Therefore, this biosensor platform is expected to further develop new applications of functionalized graphene and can be applied for clinical diagnosis in the future.

## Figures and Tables

**Table 1:** Comparison of this work with other reported cytosensors.

Modified Electrode	Detected Cells	Detection Limit (cells∙mL^−1^)	Reference
Carboxymethyl chitosan/graphene	HL-60 cells	500	[6]
folic acid conjugated graphene/hemin	HeLa cells	1000	[7]
ceramic/noble metals/3D-printed	HepG2	1000	[20]
Graphene oxide/poly-L-lysine	K_562_ cells	30	[33]
Hyaluronate/graphene	HCT-116	100	This work

## Supplementary Materials

The following are available online at http://www.mdpi.com/2072-666X/9/12/669/s1, Figure S1: Cell scratch assays. Scratch between HCT-116 cells at (a) 0 h, (b) 24 h, and (c) 48h. Scale bar 100 μm, Figure S2: △Ret response of the cytosensor: (a) bare GCE, (b) GCE incubated in 1.0 × 10^5^ cells∙mL^−1^ NIH/3T3 cells, and (c) 1.0 × 10^5^ cells∙mL^−1^ HCT-116 cells for 2 h, respectively. Error bars represent standard deviation. Every point was an average value of three models of the cytosensors for independent measurements, Figure S3: △Ret response of (a) HG and (b) NH_2_ modified GCE with 1.0×10^5^ cells∙mL^−1^ HCT-116 cells. Error bars represent standard deviation. Every point was an average value of three models of the cytosensors for independent measurements, Table S1: The advantages and disadvantages of functionalized graphene, Table S2: Fitting values of all elements in the Randle’s equivalent circuit for the different electrodes, Table S3: This work compared with other reports of detecting cancer cells.
