# Supplementary material for: Hyaluronate-Functionalized Graphene for Label-Free Electrochemical Cytosensing
Source: Micromachines (Basel). 2018 Dec 18;9(12):669. doi: 10.3390/mi9120669 (PMC6315524; doi:10.3390/mi9120669)
Supplement: Supplementary file 1 [file micromachines-09-00669-s001.pdf]

Table S1. The advantages and disadvantages of functionalized graphene.

Modified Electrode	advantages	disadvantages	Reference
Polyaniline/Graphene	Detect Mycobacterium tuberculosis， DNA biosensor	Detection limit not very low	[28]
AgNPs in PANI and N-doped graphene	Detect miRNA-21 biomarker，high sensitivity	needs further clinical validation	[29]
holey graphene	Nitrite sensor, high sensitivity, and a low detection limit	Real sample not mentioned	[30]
3D graphene/Ni3N nanoparticles	detects glucose and H_2O_2 with satisfactory selectivity, reproducibility and long-term storage stability.	Needs further electrocatalytic applications	[32]
AuNPs/rGO/thionine (THI)	detect PSA as low as 10 pg mL^{-1}, detection of clinical serum samples	Stable not mentioned	[33]
folate decorated Nitrogen-doped graphene quantum	detection of MKN 45, HT 29 and MCF 7 cancer cell	needs further clinical validation	[34]
Hollow nitrogen-doped carbon microspheres pyrolyzed from self-polymerized dopamine	simultaneous electrochemical determination of uric acid, ascorbic acid and dopamine	Detection limit not very low	[35]
Hyaluronic acid-functionalized single-walled carbon nanotubes	tumor-targeting MRI contrast agent	needs further clinical validation	[36]

Table S2. Fitting values of all elements in the Randle′s equivalent circuit for the different electrodes.

	R_s(Ω)	R_{et}(Ω)	C_{dl} (nF)	Z_w (μΩ/$s^{1/2}$)
GC	65.3 ± 4.1	164 ± 6	427 ± 22	643 ± 36
HG/GC	70.3 ± 3.9	1256 ± 48	469 ± 18	726 ± 43
HCT-116 (5.0 × 10^2 cells·mL^{-1})	73.9 ± 3.6	2436 ± 89	484 ± 23	704 ± 41
HCT-116 (5.0 × 10^3 cells·mL^{-1})	74.1 ± 3.5	2798 ± 92	476 ± 21	735 ± 30
HCT-116 (5.0 × 10^4 cells·mL^{-1})	75.2 ± 3.8	3779 ± 108	457 ± 26	698 ± 32
HCT-116(5.0 × 10^5 cells·mL^{-1})	74.7 ± 3.6	4769 ± 162	463 ± 24	734 ± 35
HCT-116 (5.0 × 10^6 cells·mL^{-1})	76.8 ± 3.7	5482 ± 211	470 ± 28	651 ± 41

Table S3. This work compared with other reports of detecting cancer cells.

Method	Detected cells	Detection limit / the concentration range	Reference
folic acid conjugation isocratic reversed-phase HPLC method	rilpivirine (HeLa cells lysate)	0.025-2 ug·mL^{-1}	[1]
liquid chromatography-tandem mass spectrometry assay (LC-MS/MS) with electrospray ionization	HepG2	of 10.0 to 10,000 ng·mL^{-1}	[2]
electrochemical	Glutathione(HeLa cells)	1.0–500.0 u M	[3]
solid phase extraction (SPE)-UPLC-MS/MS method	oroxylin A and oroxylin A 7-O-D-glucuronide (HepG2 cell lysate)	~~	[4]
Carboxymethyl chitosan/graphene	HL-60 cells	500	[5]
High Performance Liquid Chromatography Method	18-beta-Glycyrrhetinic Acid in HepG2 Cell Line	1.5-120 ug·mL^{-1} (n = 5)	[6]
Chip-based monolithic microextraction combined with ICP-MS	bismuth (HepG2 cells)	the detection limit was 0.21 ng·mL^{-1}	[7]
fluorescent probes and subsequently separated by microchip electrophoresis	superoxide and hydrogen peroxide (human HepG2 cell extracts)	~~	[8]
capillary electrophoresis	glutathione in single HepG2 cells	detection limit was 1 uM	[9]
Hydrophilic interaction liquid chromatography-tandem mass spectrometry method	intact oxaliplatin in cells	2–200 ng·mL^{-1}	[10]

Scratch test.

A scratch test was performed to assess the mobility of HCT-116 cells. In brief, HCT-116 cells were incubated in a 6-well plate, and at least five horizontal lines were drawn on the back of the plate. Then, 5 × 10^5 cells were placed in each well and cultured overnight. Straight scratches were then made vertical to the lines drawn on the back of the plate, and the wells were washed three times with PBS to remove excess cells. Cells were cultured with serum-free culture medium at 37 °C with 5% CO_2, and observed at 0 h, 12h, 24 h, and 48 h.

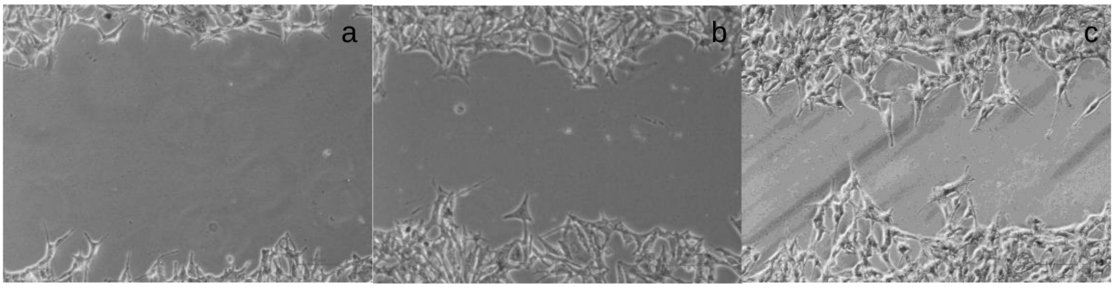

Figure S1. Cell scratch assays. Scratch between HCT-116 cells at (**a**) 0 h, (**b**) 24 h, and (**c**) 48 h. Scale bar 100 μm.

The selectivity test and the control experiments.

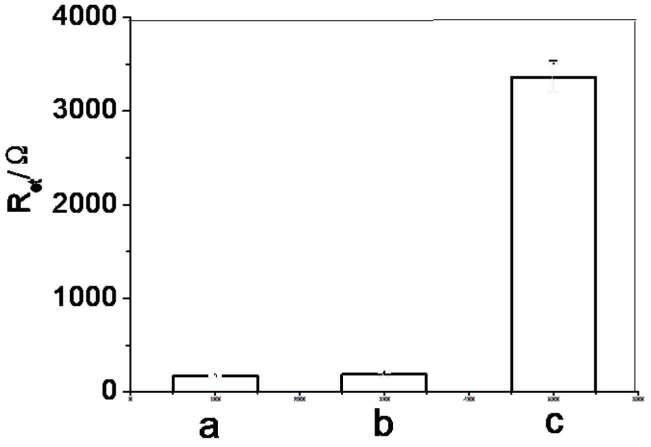

Figure S2. $\triangle R_{et}$ response of the cytosensor: (**a**) bare GCE, (**b**) GCE incubated in 1.0 × 10^5 cells·mL^{-1} NIH/3T3 cells, and (**c**) 1.0 × 10^5 cells·mL^{-1} HCT-116 cells for 2 h, respectively. Error bars represent standard deviation. Every point was an average value of three models of the cytosensors for independent measurements.

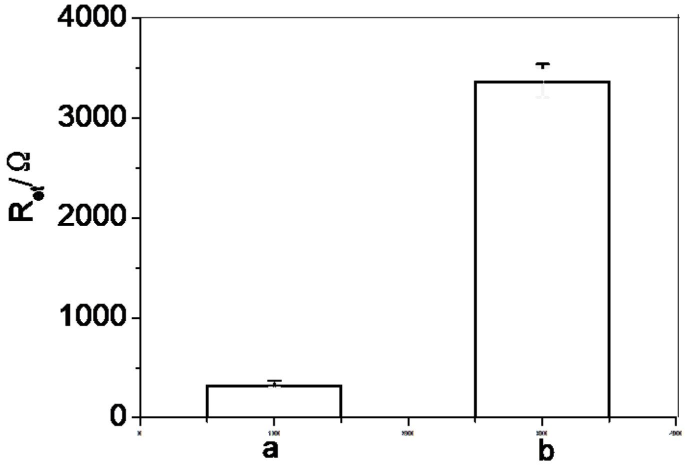

Figure S3. △R_{et} response of (**a**) HG and (**b**) NH_2 modified GCE with 1.0 × 10^5 cells·mL^{-1} HCT-116 cells. Error bars represent standard deviation. Every point was an average value of three models of the cytosensors for independent measurements.
